# Estimating Cognitive Workload in an Interactive Virtual Reality Environment Using EEG

**DOI:** 10.3389/fnhum.2019.00401

**Published:** 2019-11-14

**Authors:** Christoph Tremmel, Christian Herff, Tetsuya Sato, Krzysztof Rechowicz, Yusuke Yamani, Dean J. Krusienski

**Affiliations:** ^1^Biomedical Engineering, Old Dominion University, Norfolk, VA, United States; ^2^Department of Neurosurgery, School of Mental Health and Neurosciences, Maastricht University, Maastricht, Netherlands; ^3^Department of Psychology, Old Dominion University, Norfolk, VA, United States; ^4^Virginia Modeling, Analysis and Simulation Center (VMASC), Suffolk, VA, United States; ^5^Department of Biomedical Engineering, Virginia Commonwealth University, Richmond, VA, United States

**Keywords:** cognitive workload, electroencephalogram (EEG), virtual reality, HTC VIVE, n-back task

## Abstract

With the recent surge of affordable, high-performance virtual reality (VR) headsets, there is unlimited potential for applications ranging from education, to training, to entertainment, to fitness and beyond. As these interfaces continue to evolve, passive user-state monitoring can play a key role in expanding the immersive VR experience, and tracking activity for user well-being. By recording physiological signals such as the electroencephalogram (EEG) during use of a VR device, the user's interactions in the virtual environment could be adapted in real-time based on the user's cognitive state. Current VR headsets provide a logical, convenient, and unobtrusive framework for mounting EEG sensors. The present study evaluates the feasibility of passively monitoring cognitive workload via EEG while performing a classical n-back task in an interactive VR environment. Data were collected from 15 participants and the spatio-spectral EEG features were analyzed with respect to task performance. The results indicate that scalp measurements of electrical activity can effectively discriminate three workload levels, even after suppression of a co-varying high-frequency activity.

## 1. Introduction

The integration of user-state biofeedback to future virtual reality (VR) and augmented reality (AR) systems is vital for providing more immersive, adaptive and functional VR experiences, as well as optimizing human performance for a wide variety of application domains (Bisson et al., 2007; Lobel et al., 2016; Cipresso et al., 2018). Current VR headsets provide a logical, convenient, and unobtrusive framework for mounting EEG sensors. Additionally, recent advances in dry/wireless EEG electrodes (Wang et al., 2016; Zander et al., 2017; de Camp et al., 2018; Lee et al., 2018; Kam et al., 2019) and motion artifact suppression (Gwin et al., 2010; Daly et al., 2015; Kline et al., 2015; Snyder et al., 2015; Arad et al., 2018) further increase the practicality of integrating EEG into VR headsets.

The vast majority of literature focuses on active or reactive modulation (Zander and Kothe, 2011) of EEG to directly control or interact in the virtual environment, such as decoding imagined movement signals from EEG to navigate through the virtual environment while the user remains stationary in the physical space (Leeb et al., 2007; Scherer et al., 2008; Royer et al., 2010; Velasco-Alvarez et al., 2010; Doud et al., 2011). However, these designs often require significant user training, rely on unnatural or obtrusive sensory stimuli, and exhibit performance issues that limit practical, long-term use (Lotte et al., 2008, 2010; Ron-Angevin and Diaz-Estrella, 2009).

In contrast, implicit or passive BCI control, where the user's cognitive or affective state is passively monitored and used to affect some auxiliary aspect of the interaction (Zander and Kothe, 2011; Brouwer et al., 2015; Unni et al., 2017; Horvat et al., 2018; Ihme et al., 2018) may be better-suited for practical integration into VR systems. Such passive feedback can be designed to be less sensitive to BCI decoding errors, with the potential of being less noticeable and distracting to the user compared to decoding errors in direct BCI control of the environment. Thus, such passive feedback holds promise for improving engagement and immersion in VR.

Prior studies have attempted to classify different cognitive tasks such as rest vs. mental imagery (e.g., mental math or object rotation) using brain activity. The number of tasks and task difficulty can be altered to produce detectable changes in cognitive state. This has been effectively demonstrated in both EEG (Ruchkin et al., 1991; Ryu and Myung, 2005; So et al., 2017) and fNIRS (Power et al., 2010, 2012; Herff et al., 2013b). Other studies have further investigated changes in brain activity with respect to changes in cognitive workload during task performance using EEG (Berka et al., 2007; Brouwer et al., 2012; Gerjets et al., 2014; Hogervorst et al., 2014; Mühl et al., 2014; Ewing et al., 2016; Schultze-Kraft et al., 2016; Grissmann et al., 2017a,b; Scharinger et al., 2017; Pergher et al., 2018) and fNIRS (Ayaz et al., 2012; Herff et al., 2013a; Unni et al., 2017). It has also been shown that cognitive workload models trained on one task condition can be effectively transferred to other conditions (Baldwin and Penaranda, 2012).

Studies have also used passive neurofeedback of EEG or fNIRS to modulate the controllability of the player's avatar in a video game (Muhl et al., 2010), the transformation of the avatar into another physical form (Bos et al., 2010), the adaptation of the game difficulty (Girouard et al., 2013), or to monitor items in the VR environment that were detected by the user (Zander et al., 2010). See Lécuyer et al. (2008), Lotte et al. (2013), and Kerous et al. (2018) for reviews of the application of brain-computer interfaces for VR and videogames.

The aforementioned EEG-based studies largely utilize the various combinations of the traditional power spectral bands: θ, α, β, γ over frontal, central, and parietal locations. Of particular relevance to estimating cognitive workload and working memory from EEG, numerous studies have indicated that the fronto-parietal network exhibits a decrease in α power with increasing task demands, while θ power is positively correlated with increasing task demands (Sauseng et al., 2005, 2010; Brouwer et al., 2012). Other studies suggest that β activity behaves similarly to α, but may be due to motor activity required by the tasks (Pesonen et al., 2007; Scharinger et al., 2017). Due to the limitations of scalp EEG, γ activity has been less frequently reported in relation to cognitive workload. Fitzgibbon et al. found widespread γ activations in a variety of cognitive tasks (Fitzgibbon et al., 2004). Tallon-Gaudry et al. revealed a specific γ-band feature for a memory task that appeared decoupled from head and neck muscle activity (Tallon-Baudry et al., 1998). Additionally, magnetoencephalographic (MEG) and electrocorticographic (ECoG) indicate that α-γ and θ-γ coupling play a role in working memory (Roux and Uhlhaas, 2014).

**Figure 1:**
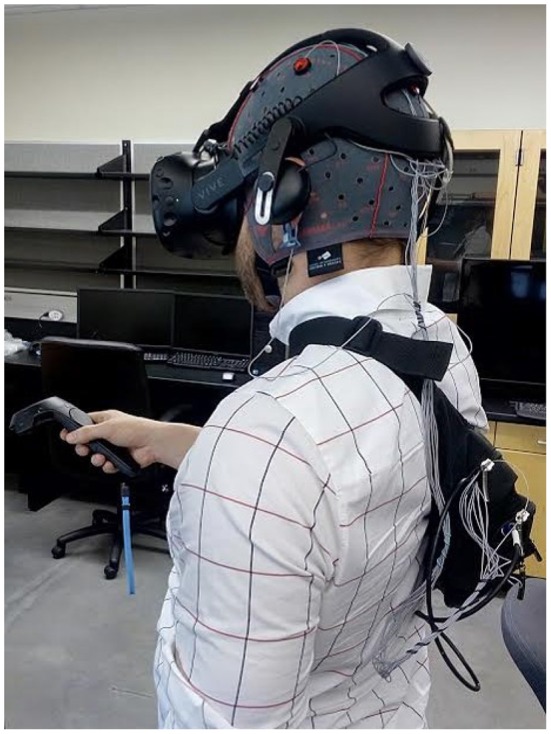
Configuration of the experimental equipment on a participant (excluding the protective plastic hair dressing cap).

The present study aims to build upon the prior work on passive EEG-neurofeedback using the *n*-back task (Brouwer et al., 2012) through the use of an interactive, head-mounted VR experience. This represents a deliberate attempt to move beyond controlled and sterile experimental environments toward a practical VR application, where there is a detailed and potentially-distracting environment where the user is physically interacting with objects to perform a task. In order to verify that EEG measures of cognitive workload can be reliably attained using an interactive VR environment, we adapted the well-established *n*-back task (Kirchner, 1958) for modulating cognitive workload into an immersive virtual environment using a HTC VIVE VR headset^1^. For the classical *n*-back task, participants are presented with a series of symbols and are asked to respond when the current symbol matched the symbol presented *n* symbols ago in the sequence. The cognitive workload increases as a function of increasing *n*. To adapt the task to a more immersive, game-like virtual environment, stimuli were a series of colored balls presented on a virtual podium the VR headset as shown in Figure 1.

The details of the environment were intentionally designed to be video game-like to increase the level of immersion for comparison of task performance to prior, less visually-distracting desktop-based studies. While the present study does not implement closed-loop BCI control, the intention is to inform the integration of EEG-based feedback into future interactive VR systems.

## 2. Materials and Methods

### 2.1. Participants and Experimental Setup

Fifteen participants [ages 18–35 (mean 24.73), 4 female, all right-handed] were recruited to participate in the experiment, which was approved by the Institutional Review Board of Old Dominion University. Participants first completed an informed consent, a visual acuity test, the Motion Sickness Susceptibility Questionnaire short-form (MSSQ-short; Golding, 2006), and the Ishihara Color Blindness test (Clark, 1924). All participants tested satisfied the inclusion criteria, specifically, all participants read the 20/30 line on the visual acuity test, scored at least 19 on the MSSQ, and correctly determined all symbols on the color-blindness test.

The HTC VIVE hardware system primarily consists of a motion-tracked headset display, two motion-tracked hand controllers, and two “lighthouse” base stations that are capable of providing 6 Degree of Freedom (6DOF) tracking. After the screening process, the EEG cap was placed on the participant's head and the EEG electrodes were filled with electrolyte gel. The electrode cap was then covered with a protective plastic hair dressing cap to insure that the gel did not seep onto the VR headset, and the VR headset was positioned over the EEG cap. The wireless EEG amplifier was placed in a shoulder strap on the participants back. The configuration of the experimental equipment on a participant (excluding the protective plastic hair dressing cap) is shown in Figure 1.

After the EEG and VR equipment was positioned, participants grasped a VIVE hand controller in the dominant hand (i.e., the right hand for all participants). Participants were placed in a standing position approximately 1 m in front of the recording computer, within the VR workspace.

### 2.2. Experimental Task

**Figure 2:**
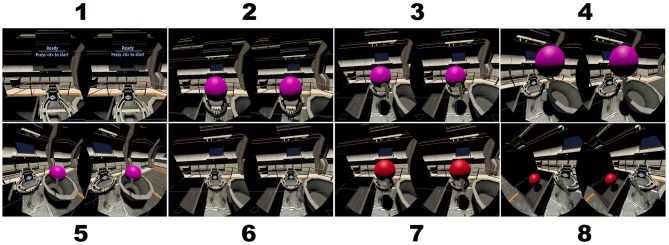
Screen captures of a single trail of the n-back task using colored balls in the interactive virtual environment. Each frame represents the binocular view as observed through the VR headset. (1) The podium and instruction display. (2) The trial begins when the colored ball appears. (3) Participant uses the trigger on the hand controller to grasp the ball. (4) Participant moves the ball to the right toward the non-target receptacle. (5) Participant releases the ball in the non-target receptacle and the trial ends. (6–8) A new trial begins for which the ball is placed in the target receptacle to the left.

Stimuli are a series of colored balls presented on a virtual podium the VR headset. Following McMillan et al. (2007), each ball is colored red, blue, purple, green, or yellow. A ball receptacle is placed to the right and left of the participant in the virtual environment. The target receptacle was shaped as treasure chest. For a particular run, the participant's task was to pick up a virtual ball from the podium directly in front of them using the hand controller and move it to the target receptacle if the current ball color matched the color of the ball presented N trials before and to the opposite receptacle otherwise. Screen captures illustrating a single trial of the task are shown in Figure 2.

Participants completed a 5 min practice block to familiarize themselves with the VR system and the *n*-back task. For the practice block, participants performed the 1-back task until one run consisting of a random sequence of 20 balls was completed without any errors. Following the practice block, participants performed a series of three experimental blocks in randomized order: 0-back, 1-back, and 2-back blocks consisting of 4 runs each. For the 0-back task, participants simply determine whether each ball is red or not.

For each block, participants received specific instructions regarding the task, followed by 4 experimental runs of the same n-back task. Each experimental run consisted of a random sequence of 20 balls, each of them remaining visible for 4 s, immediately followed by the onset of the next ball. Only a single ball is displayed at any given time and an auditory tone signaled the appearance of each new ball. The sequence of ball colors was generated randomly such that a minimum of 2 target trials were present in the run. The empirical maximum number of targets in a run was 7.

Participants were required to respond to all balls in each experimental run. Failure to respond (i.e., not placing the ball in a receptacle before the end of the trial) reset the run from the beginning and negated the erroneous run. While such run resets occurred for several participants during the training run, only a single reset for a single participant occurred during the actual experimental runs.

The order of the experimental blocks were counterbalanced across participants. For each participant, the target receptacle locations were counterbalanced to avoid biases that may be created by the lateral movements. To help engage participants, the performance (percent correct) was displayed after each trial. The total duration of the experiment was kept to 20 min to reduce the risk of simulator sickness, thus the time between successive runs and blocks was less than a minute.

### 2.3. Data Collection and Analysis

**Figure 3:**
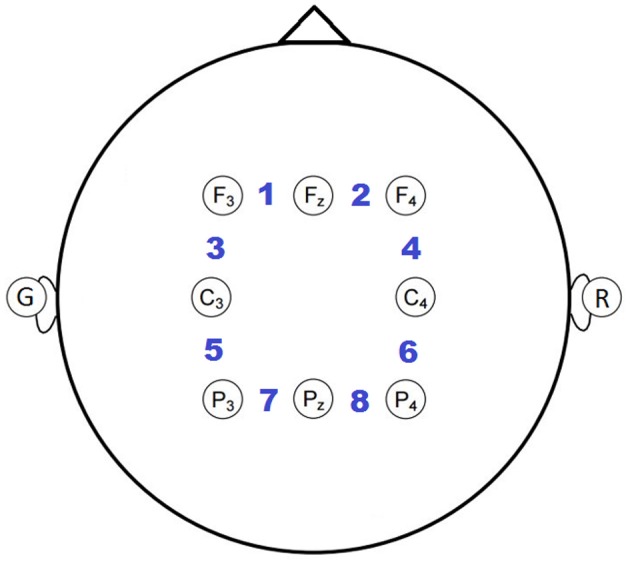
Electrode montage with bipolar channels indicated by the numbering between adjacent electrode pairs.

Each participant wore an 8-channel electrode cap (g.LADYBIRD, Guger Technologies) with active electrodes positioned based on the international 10-20 system (Sharbrough et al., 1991). Specifically, electrode positions F3, Fz, F4, C3, C4, P3, Pz, P4 were used (see Figure 3), based on neural activations from prior EEG and fMRI studies (Owen et al., 2005; Brouwer et al., 2012). EEG was collected using an 8-channel wireless biosignal amplifier (g.MOBIlab, Guger Technologies), grounded to the left earlobe, referenced to the right earlobe, and digitized at a 256 Hz.

The position of the VR headset and the controller were also tracked and digitized at 32 Hz. Communication between the VR software (developed in Unity^2^) and the BCI2000 EEG recording software was performed via UDP communication using the application connector in BCI2000 (Schalk et al., 2004).

A bipolar reference was applied because it empirically minimized the correlation of high frequency activity, presumed to be due primarily to scalp muscle tension (e.g., frontalis, temporalis, and/or occipitalis), with the task compared to an ear or common-average reference. Eight bipolar channels were created by subtracting the adjacent earlobe-referenced channels from right to left and anterior to posterior, as indicated by the numbered positions in Figure 3.

A conservative Hampel outlier filter was applied to the EEG reduce the occasional impulse-like artifact due to the wireless transmission. The Hampel filter computes the median of a sliding 1-second window centered on the current sample. The median absolute deviation is computed over the window. If the current sample differs from the median by more than five standard deviations, it is replaced with the median. The processed EEG was visually inspected to verify the efficacy of the artifact removal.

The EEG data were segmented by 4-second ball-presentation intervals (i.e., trials), yielding 240 total trials (4 runs × 3 conditions × 20 balls per run) per participant. The last trial of each run was excluded from the analysis due to a software issue that prematurely terminated data collection, which resulted in 228 total trials per participant for analysis. Because task performance satisfactory for all participants (see Results section), all trials (i.e., correct and incorrect ball placements) were included in the analysis.

The frequency spectrum of the EEG was computed for each 4 s run using Welch's method with a 256-point FFT and 50% overlap. The resulting spectral amplitudes were log transformed and the spectral bins were averaged over the traditional EEG bands: θ (5–7 Hz), α (8–14 Hz), β (15–30 Hz), and γ (31–55 Hz). Frequencies below 5 Hz are prone to gross movement artifacts and were excluded from the analysis. Additionally, a higher frequency range termed *HF* (70–100 Hz) was analyzed. This band was shown to be correlated with the task and is outside the frequency range of typical scalp EEG, thus the task-dependent variations of this band are suspected to be modulated in-part by subtle scalp muscle tension.

The data were parsed by *n*-back level. To explore the univariate characteristics of each spectral feature, Spearman's correlation was computed between the *n*-back level and the spectral amplitude for each frequency band and bipolar channel. Because the HF band exhibited large task-related correlations relative to the lower-frequency bands, it is suspected that this high-frequency band is due in-part to task-dependent EMG resulting from scalp muscle tension as suggested in Mühl et al. (2014). Since EMG activity is broadband and likely also pervades the low-frequency bands (Goncharova et al., 2003; Fu et al., 2006; Muthukumaraswamy, 2013; Yilmaz et al., 2014; Janani et al., 2017), a linear regression model was applied to reduce the correlation of this high-frequency activity in the lower frequency bands. Using the trial-wise spectral amplitude of each lower frequency band as the regressand (i.e., θ, α, β, and γ), a linear regression model was generated with the corresponding HF-band spectral amplitude as the regressor. The model was then used to remove the correlated activity by subtracting the model output from the respective regressand. This approach is referred to herein as HF suppression.

Spearman's correlation was used to quantify the relationship between the univariate spatio-spectral features and the cognitive workload level. To explore the multivariate discriminative power of the spatial and spectral features, various combinations of the features (5 spectral bins X 8 channels) were classified using regularized linear discriminant analysis (rLDA) with a four-fold cross-validation (due to the number of trials being perfectly divisible by 4). Specifically, the HF suppression (i.e., regression) approach was applied to the training data for each fold and the *fitcdiscr* function in MATLAB was used to preform the rLDA and optimize the regularization parameters. The HF suppression was performed separately on the test data for each fold.

## 3. Results

The average task performance (correct bin placement) was 99.67±1.56% for *n* = 0; 98.17±4.51% for *n* = 1; and 95.83±7.49% for *n* = 2. Fourteen participants scored above 80% on all runs; the remaining participant scored above 70% on all runs. Twelve of the participants scored above 90% on all runs.

**Figure 4:**
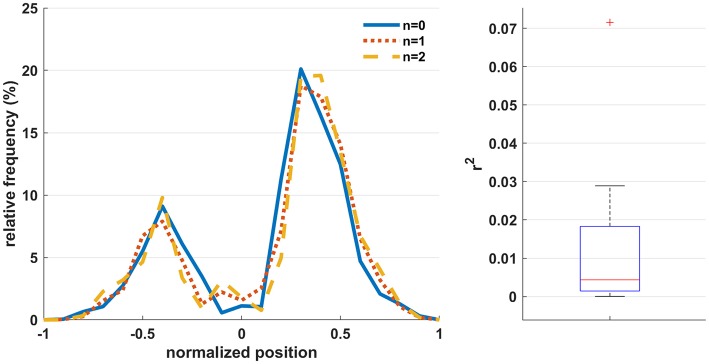
**(Left)** The relative frequency of the normalized hand-controller position (computed as the signed resultant of the horizontal x-y hand-controller coordinates) across participants for the three workload conditions. Positive and negative values indicate positions to the right and left of center, respectively. **(Right)** The r-squared correlation of the workload level with the normalized hand-controller position across participants.

Figure 4 (left) shows the relative frequency of the normalized hand-controller position (computed as the signed resultant of the horizontal x-y hand-controller coordinates) across participants for the three workload conditions. It is observed that the distribution of controller positions is consistent across conditions. The rightmost peak is larger due to the fact that all participants were right-handed and the base position is right-of-center. Figure 4 (right) shows a boxplot^3^ of the r-squared correlation of the workload level with the normalized hand-controller position across participants, indicating that the movements exhibit minimal bias with respect to the workload level.

**Figure 5:**
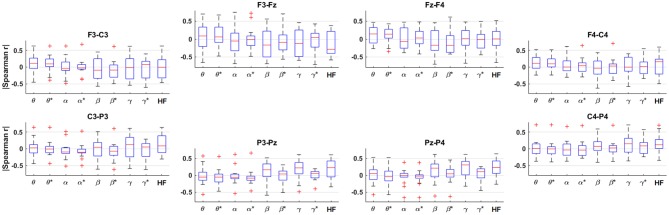
Box plots across participants of the Spearman correlation between the spectral amplitude and workload level for each frequency band and bipolar channel, arranged topographically by channel. The title of each subplot indicates the polarity of the bipolar channel. The asterisks (*) indicate the result after HF suppression.

The results of the Spearman correlation analysis for each band and bipolar channel are shown in Figure 5, where the HF suppression is indicated with asterisks (*). Note that the HF band exhibits correlation values in the same general range as the traditional EEG bands workload level for most channels, and that the magnitude of the correlations in each band across participants is somewhat inconsistent in the frontal channels and more consistent in the posterior channels. It can also be observed that the magnitude of the correlations generally drop by varying degrees in each frequency band after HF suppression.

**Figure 6:**
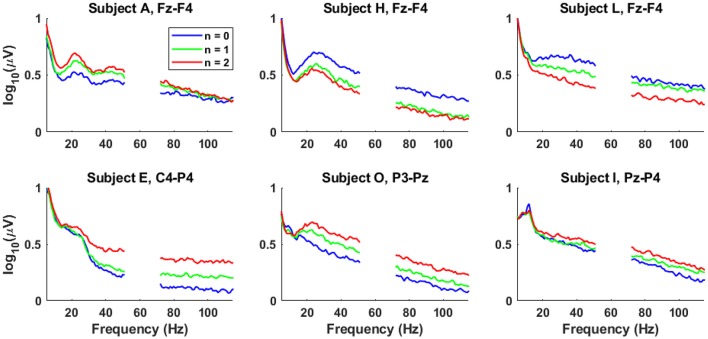
Selected log-amplitude spectra from six different participants across workload levels. The top row represents frontal channels and the bottom row represents posterior channels.

The differences in average spectral amplitude across conditions for selected participants and channels are shown in Figure 6. While there are clear broadband differences across workload levels for particular channels and some common activity across subsets of participants (i.e., participants H and L in Figure 6), it should be noted that neither the channels nor the relative spectral amplitude modulation across workload levels (i.e., participants A and H/L in Figure 6) appear consistent across participants.

**Figure 7:**
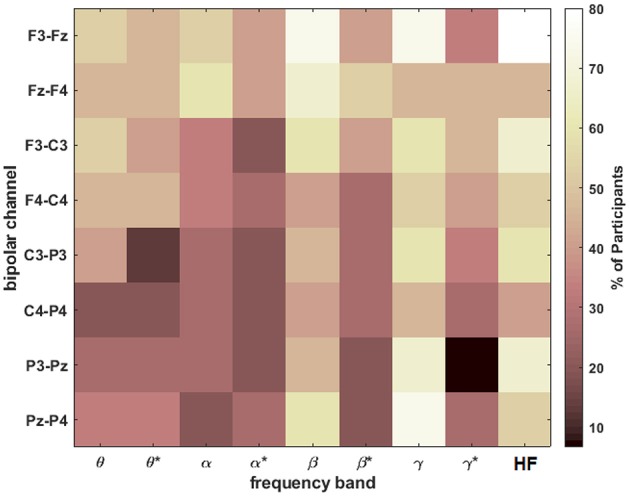
The percentage of participants with statistically significant differences (after Bonferroni correction) in spectral amplitude between *n* = 0 and *n* = 2 for each band and bipolar channel. The asterisks (*) indicate the result after HF suppression.

To assess the most discriminable univariate features across participants, a two-sided Wilcoxon rank sum test was used to determine the percentage of participants with statistically-significant differences in spectral amplitude between the extreme workload levels of *n* = 0 and *n* = 2 for each feature. The results shown in Figure 7 were Bonferroni corrected to a significance level of 6.94 × 10^−4^ [0.05/(9 frequency bands × 8 channels)].

The HF band at F3-Fz was significant for 80% of the participants. Otherwise, the most consistent features for roughly 73% of the participants were β and γ at F3-Fz and γ at Pz-P4, all before HF suppression. After HF suppression, multiple feature have roughly 50% prevalence including frontal/central θ, β at Fz-F4, and γ at Fz-F4/F3-C3.

**Figure 8:**
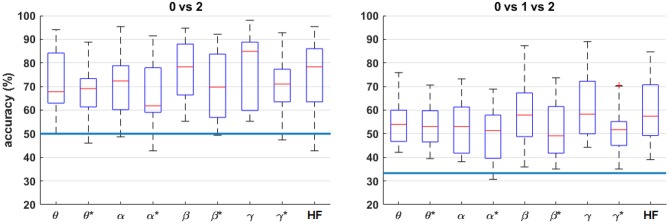
Box plots of the four-fold classification accuracy across participants for each combination of workload levels. For each frequency band, all bipolar channels were included as features for the classifier. The blue horizontal lines indicate the chance level of classification accuracy. The asterisks (*) indicate the result after HF suppression.

Figure 8 shows the four-fold classification accuracy for each frequency band using all bipolar channels for *n* = 0 vs. 2 and *n* = 0 vs. 1 vs. 2. Similar to the correlation analysis, the HF band achieves a comparatively high classification accuracy, and the performance generally drops for each band after HF suppression. However, the inter-quartile range of each comparison is well above the random-chance level, even after HF suppression.

**Figure 9:**
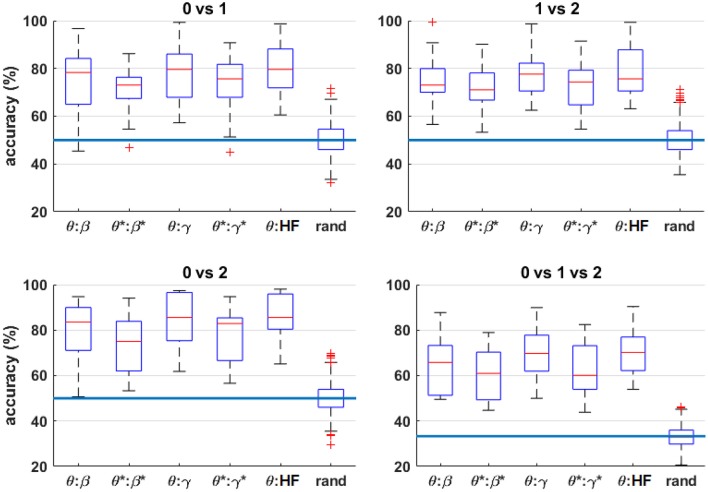
Box plots of the four-fold classification accuracy across participants for each combination of workload levels. The horizontal axis indicates the range of frequency bands included in the classifier. The blue horizontal lines indicate the random-chance level of classification accuracy. The “rand” label indicates the classification results from randomly permuting the labels for the θ:HF features, which produces nearly identical results for all feature combinations tested. The asterisks (*) indicate the result after HF suppression.

**Figure 10:**
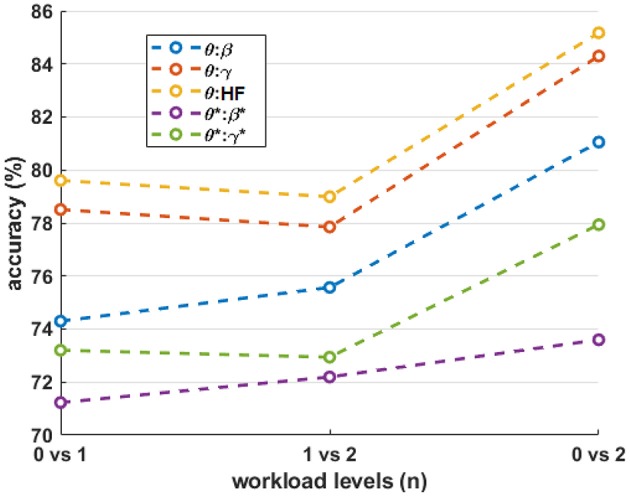
The mean accuracy of the various workload level comparisons for different frequency-band combinations. The asterisks (*) indicate the result after HF suppression.

Figure 9 shows the four-fold classification accuracy using all bipolar channels and various frequency ranges as features for the classifier. To further indicate the significance of the classification results, permutation tests were performed by randomizing the class labels for each scenario, performing the classification procedure, and repeating 100 times. Since the results were nearly identical for each feature combination for a given workload-level comparison, the random permutation results for the θ:HF condition are included in the figure, labeled as “rand.” Similar to the results shown in Figure 8, HF suppression decreased performance for all conditions and the inter-quartile range of each condition are above inter-quartile range randomization test. For *n* = 0 vs. 1 vs. 2, all observations are above inter-quartile range randomization test. Figure 10 shows the average accuracy trends across the workload level comparisons for different frequency-band combinations.

**Figure 11:**
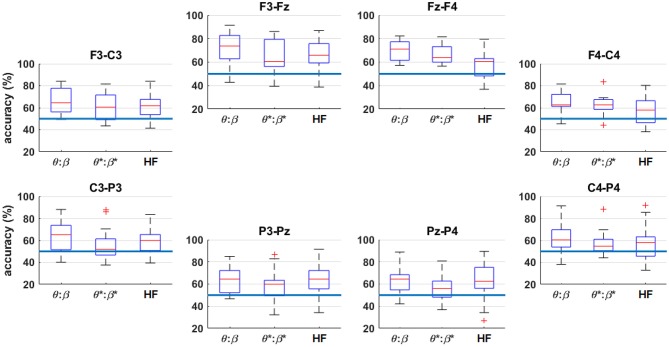
Box plots of the four-fold classification accuracy across participants for *n* = 0 vs. *n* = 2, arranged by channel. The horizontal axis indicates the range of frequency bands included in the classifier. The blue horizontal lines indicate the random-chance level of classification accuracy. The asterisks (*) indicate the result after HF suppression.

To further examine the spatial contributions of the multi-band classification, Figure 11 shows the four-fold classification accuracy workload level extremes of *n* = 0 vs. *n* = 2, arranged by channel. For each channel, the traditional low-frequency EEG bands (θ to β), and HF for comparison, were included as features for the classifier. The inter-quartile ranges are generally higher in the frontal channels compared to the parietal channels. The effects of the HF band are most prominent on the left frontal channel (F3-Fz) and the posterior channels (P3-Pz and Pz-P4).

## 4. Discussion

The results of this study demonstrate that it is possible to discriminate several mental workload levels using electrical activity recorded from the scalp during an interactive VR task. Figure 7 indicates that the most consistent features across participants are frontal β and γ and parietal γ prior to HF suppression. However, after HF suppression, frontal β and γ are the most consistent features across participants, though appreciably less consistent compared to the un-corrected features.

Further examining the univariate features in Figure 5, the parietal activity above the α band is generally positively correlated with workload level. Otherwise, there is high variability across participants, which is consistent with that reported in Brouwer et al. (2012). However, the classical fronto-parietal α/θ activations (Sauseng et al., 2005, 2010; Brouwer et al., 2012) were not consistently observed across participants in the present study. Additionally, β/γ were more prominent in the present study compared to prior findings. These differences may be due to the fact that the present study used an interactive, immersive VR design that incorporated stereotyped movements and rich visual input compared to prior related studies. For example, it has been reported that VR generated more β/γ activity compared to the real-world medium in hand illusion experiments (Škola and Liarokapis, 2016). Another study that analyzed EEG collected during an interactive VR stepping game found statistically-significant hemispherical differences in the α, β, γ bands (de Oliveira et al., 2018).

The HF band exhibits comparable correlations to the lower frequencies in nearly all channels. The positive central/parietal correlations is consistent with broadband EMG due to subtle scalp muscle tension (Goncharova et al., 2003; Fu et al., 2006; Janani et al., 2017). However, Figures 5, 6 suggest that the high-frequency activity in the frontal channels is not always positively correlated with task difficulty, which is not indicative of consistent task-related muscle tension and may be due to arbitrary, spontaneous muscle activity—possibly linked to the head-mounted display.

The single frequency-band classification accuracy in Figure 8 shows that most frequency bands exhibit roughly similar performance ranges before and after HF suppression, respectively. As expected, this can generally be extended to the combined-band results in Figure 9, with the combined-band results generally exhibiting higher overall accuracy ranges. This suggests that spectral bands contain some degree of complimentary information for classification. As shown in Brouwer et al. (2012), Figure 10 reaffirms that the extreme workload levels (i.e., 0 vs. 2) are more clearly discriminable that the first-degree levels of 0 vs. 1 and 1 vs. 2, with 0 vs. 1 being the least discriminable.

Figure 11 shows that the frontal channels tend to produce higher classification accuracies. When assessing the spatial (Figure 11) and spectral (Figure 8) contributions in isolation it is noted that, in general, there are not drastic differences in the performances across bands or channels. However, by comparing to the combined-band results of Figure 9, information from multiple frequencies and channels is crucial for maximizing performance.

This result may be a function of unique and complementary information in the various channels and frequency bands, but it may also be an indication of individual differences as suggested in Figures 5, 6, and in Brouwer et al. (2012). While Figure 7 appears to indicate prevalent features across participants, in a 15-fold transfer learning protocol (training on all combinations of 14 participants and testing on the remaining participant), the training error was high and the cross-validation results were only slightly above random chance - further supporting the notion of individual differences across participants. Possible explanations for the individual differences could be due to varying memory span, cognitive ability (Gonzalez, 2005), or arousal (Matthews and Davies, 2001); vigilance decrement (Mackworth, 1968; Warm et al., 2008); or fatigue effects over the duration of the task.

Further analysis indicated that a straightforward 5 Hz highpass filter is effective at suppressing low-frequency artifacts due to the deliberate, stereotyped gross movements required for the task. However, the elimination of EMG artifacts due to more subtle scalp tension remains a significant challenge (Goncharova et al., 2003; Fu et al., 2006; Muthukumaraswamy, 2013; Yilmaz et al., 2014, 2018; Janani et al., 2017). Because of the overlapping frequency ranges, it is effectively impossible to definitively isolate EMG and EEG activity without applying a neuromuscular blockade (Whitham et al., 2008). Furthermore, studies suggest that any degree of cognitive workload will create subtle, correlated head and neck muscle tension that further confounds such analysis (Laursen et al., 2002; Krantz et al., 2004; LC Leyman et al., 2004; Whitham et al., 2008; Roman-Liu et al., 2013).

The present approach uses linear regression to remove the correlated activity of a high-frequency band from the low-frequency EMG bands. While this may generate a reasonable approximation of EMG activity, there are several issues with this simplistic approach. Firstly, this approach assumes that there is a linear relationship between the dynamics of EMG contamination across frequency bands (Kim et al., 2017). If this relationship is not linear, then residual EMG artifact will be present in the EMG-suppressed signal. Secondly, assuming the high-frequency EMG activity is highly-correlated with the task, this regression approach may be suppressing genuine EEG relationships with the task (Mühl et al., 2014). Overall, these two contrasting effects may cancel to a degree and result in a reasonable estimate of the lower-frequency EEG activity.

In summary, this analysis demonstrates that cognitive workload during an interactive VR task can be estimated via scalp recordings. Using the traditional low-frequency EEG bands (θ−β), average workload classification accuracies across reached 81.1% (chance 50%) for 0 vs. 2 and 63.9% (chance 33.3%) for 0 vs. 1 vs. 2. By comparison, classification accuracies of 73.6 and 60.6%, respectively, can be achieved using the same bands after HF suppression. The recordings appear robust to the head-mounted setup and gross-movements. However, the results suggest that the cognitive workload task generates individual differences in brain activity, which likely require the development of subject-specific models. Furthermore, there are likely contributions of both EEG and scalp muscle tension-related EMG to cognitive workload classification. Ultimately, for practical cognitive workload discrimination, it may not be necessary to isolate EEG from EMG if the result is effective. However, in this case, care must be taken such that EMG does not become consciously or subconsciously conditioned to be predominant over EEG for manipulating the task outcome in closed-loop scenarios, as EEG (or other measures of brain activity) represents the intrinsic biomarker of cognitive workload.

## Data Availability Statement

The experimental software used for this study is available in The Open Science Framework (https://osf.io/yhtz8/). The data generated for this study is available upon request to the corresponding author.

## Ethics Statement

This study was carried out in accordance with the recommendations of the Institutional Review Board of Old Dominion University with written informed consent from all subjects. All subjects gave written informed consent in accordance with the Declaration of Helsinki. The protocol was approved by the Institutional Review Board of Old Dominion University.

## Author Contributions

CH, KR, YY, and DK developed the experimental design and protocol. CT and KR configured the experimental setup. CT and TS collected the experimental data. CT and DK performed the data analysis and wrote the original draft of the manuscript. All authors contributed to reviewing and editing of the manuscript.

### Conflict of Interest

The authors declare that the research was conducted in the absence of any commercial or financial relationships that could be construed as a potential conflict of interest.

## Footnotes

^1^https://www.vive.com/

^2^https://unity3d.com/

^3^Boxplots were generated using the ‘*boxplot* function in MATLAB, where the central mark indicates the median, and the bottom and top edges of the box indicate the 25th and 75th percentiles, respectively. The whiskers extend to the most extreme data points and outliers are indicated with the “+” symbol.
